# WHEN CONTEXT GETS UNDER THE SKIN: HOW ATTITUDES SHAPE CONTEXTUAL IMPACT ON AGING WELL IN LATE LIFE

**DOI:** 10.1093/geroni/igae098.0280

**Published:** 2024-12-31

**Authors:** Oliver Schilling, Björn Slaug, Oliver Schilling

**Affiliations:** Chair; Co-Chair; Discussant

## Abstract

In late life, increasing risks of loss of resources may aggravate vulnerability to contextual conditions that challenge habitual life conduct. However, evidence of older adults’ capabilities to deal with adversity suggests that subjective accounts of enabling or challenging contexts and corresponding attitudes, rather than objective context per se, shape conditions for aging well. This symposium highlights recent findings from studies that examined the role of subjective attitudes in associations between contextual conditions and aging well in very old adults: Sabatini et al. analyze associations between changes in depressive symptoms, attitudes to aging, and indicators of aging well (life satisfaction, informant-rated disability) in an Australian sample, their findings suggest a crucial role of attitudes to aging in mediating effects of poor mental health on aging well. Slaug et al. show that effects of functional limitations on active aging were moderated by how older Swedes perceived their housing (i.e., meaning of home). Zimmermann & Demirer show that impacts of neighborhood socioeconomic deprivation in Germany on functional health were mediated by social cohesion (i.e., trusting people in the neighborhood). Using multiple German very old samples, Kaspar et al. propose and test how a contextually enriched model of successful aging, comprising perceptions of contextually driven opportunity structures could better account for the realities of aging well with disability. Schilling will discuss these findings, focusing on needs to understand the interplay between subjective attitudes, contexts and aging well in late life.

